# The role of musical training in emergent and event-based timing

**DOI:** 10.3389/fnhum.2013.00191

**Published:** 2013-05-14

**Authors:** L. H. Baer, J. L. N. Thibodeau, T. M. Gralnick, K. Z. H. Li, V. B. Penhune

**Affiliations:** Department of Psychology, Centre for Research in Human Development, Concordia UniversityMontréal, QC, Canada

**Keywords:** music, timing, finger tapping, circle drawing, emergent timing, event-based timing

## Abstract

**Introduction:** Musical performance is thought to rely predominantly on event-based timing involving a clock-like neural process and an explicit internal representation of the time interval. Some aspects of musical performance may rely on emergent timing, which is established through the optimization of movement kinematics, and can be maintained without reference to any explicit representation of the time interval. We predicted that musical training would have its largest effect on event-based timing, supporting the dissociability of these timing processes and the dominance of event-based timing in musical performance.

**Materials and Methods:** We compared 22 musicians and 17 non-musicians on the prototypical event-based timing task of finger tapping and on the typically emergently timed task of circle drawing. For each task, participants first responded in synchrony with a metronome (Paced) and then responded at the same rate without the metronome (Unpaced).

**Results:** Analyses of the Unpaced phase revealed that non-musicians were more variable in their inter-response intervals for finger tapping compared to circle drawing. Musicians did not differ between the two tasks. Between groups, non-musicians were more variable than musicians for tapping but not for drawing. We were able to show that the differences were due to less timer variability in musicians on the tapping task. Correlational analyses of movement jerk and inter-response interval variability revealed a negative association for tapping and a positive association for drawing in non-musicians only.

**Discussion:** These results suggest that musical training affects temporal variability in tapping but not drawing. Additionally, musicians and non-musicians may be employing different movement strategies to maintain accurate timing in the two tasks. These findings add to our understanding of how musical training affects timing and support the dissociability of event-based and emergent timing modes.

## Introduction

The production of accurately and precisely timed movement is a key aspect of many activities. Many forms of musical performance, such as drumming in a jazz ensemble, are characterized by mostly discrete movements with explicit start and stop events. This kind of behavior is thought to generally rely on event-based timing, which involves a clock-like neural process and an explicit internal representation of the time interval (Wing and Kristofferson, 1973). In contrast, other activities, such as the laboratory task of repetitive, continuous circle drawing, are characterized by smoothly produced movement and are thought to normally engage emergent timing in which timing can be maintained without reference to any explicit representation of the time interval (Turvey, 1977; Robertson et al., 1999). It has been proposed that event-based and emergent timing are dissociable systems, both cognitively (Zelaznik et al., 2002; Zelaznik and Rosenbaum, 2010; Delignières and Torre, 2011) and neurophysiologically (Spencer et al., 2003). However, the circumstances under which a given timing mode is engaged are less clear, with recent studies showing that tasks typically thought to use event-based timing can exhibit emergent timing behavior and vice versa (Studenka and Zelaznik, 2008; Zelaznik and Rosenbaum, 2010; Delignières and Torre, 2011). Musicians are known to excel at event-based timing tasks (Franěk et al., 1991; Collier and Ogden, 2004; Repp, 2005, 2010; Repp and Doggett, 2007; Bailey and Penhune, 2012) but, to the best of our knowledge, musicians and non-musicians have never been compared on both event-based and emergent timing tasks. It may be the case that some musical performance, such as the movement involved in controlling a violin bow, involves emergent timing and, therefore, the skills gained by musicians via years of practicing timing tasks may improve both event-based and emergent timing. In the present study, we compared musicians and non-musicians on both finger tapping and circle drawing, the prototypical event-based and emergent timing tasks respectively. We predicted that musical training would have its largest effect on event-based timing behavior, supporting the dissociability of these timing processes and the dominance of event-based timing in musical performance, and informing us as to the limits of transferability of musical skill.

The experimental paradigm most often used to study event-based timing is the finger tapping task (Repp, 2005). Participants first tap in synchrony with an auditory metronome (Paced phase); when the metronome stops, they are asked to continue tapping at the same rate (Unpaced phase). Wing and Kristofferson (1973) proposed the classic model of event-based timing for the Unpaced phase of the task, in which participants presumably rely on an internal timing process in the absence of external cues. The model assumes a central stochastic timer operating independently of the motor response and partitions the variability of the inter-response interval (IRI) into timer and motor sources. Timer variability increases linearly with mean IRI while motor variability is independent of the IRI (Wing, 2002). It has also been shown that lesions to lateral cerebellar regions increase timer but not motor variability, while medial cerebellar lesions have the converse effect (Ivry et al., 1988). Overall, there is extensive empirical support for the independence of timer and motor processes in the Wing-Kristofferson model (for full review see Wing, 2002).

The effects of musical expertise on event-based timing have been well studied. Musicians exhibit greater accuracy and less variability in both the Paced and Unpaced phases of the tapping task compared to non-musicians (Franěk et al., 1991; Repp, 2005, 2010; Repp and Doggett, 2007). Collier and Ogden (2004) developed an extension to the Wing-Kristofferson model that accounts for clock drift and showed that musical experience is related to lower motor variance, clock variance and clock drift.

In contrast to event-based finger tapping, the task that has most commonly been used to investigate emergent timing is continuous circle drawing. In the Paced phase of this task, participants continuously trace a circle in time with a metronome such that on each cycle, the drawing instrument must pass through an anchor point in synchrony with the metronome. In the Unpaced phase, participants keep drawing at the same rate but without a metronome. Evidence for a separate “emergent timing” system based on circle-drawing results was first put forward by Robertson et al. (1999), who showed that intra-individual variability of timing on a tapping task was unrelated to timing variability on the circle drawing task. These results indicated that tapping and circle drawing engaged different timing processes, and it was suggested that the class of movement, discrete or smoothly produced, determined which process was engaged.

In emergent timing, the target interval has no explicit internal representation. Instead, timing is thought to emerge from the kinematics of the required movements (Turvey, 1977; Robertson et al., 1999). For example, in the case of continuous circle drawing, a kinematic profile will be established after the first few iterations. This may be manifested by minimal cycle-to-cycle variability in acceleration or some other kinematic parameter and will presumably also be evident in the establishment of patterns of physiological measures such as muscle activation in the drawing hand. Timing, which begins as a constraint for the optimization of kinematics, becomes an epiphenomenon or an emergent property of the kinematics once optimization is achieved.

Continuous circle drawing performance has been shown to be similar to finger tapping performance for the initial few cycles only, presumably when kinematic parameters are still being stabilized (Zelaznik et al., 2005). It has also been shown that the temporal variability of intermittent circle drawing is related to that of tapping but not to that of continuous circle drawing, suggesting that differences in timing performance are not due to differences in task complexity (Zelaznik et al., 2002). Furthermore, cerebellar lesions disrupted timing in finger tapping but not circle drawing (Spencer et al., 2003). An fMRI study of discrete versus smoothly produced air tapping found that the cerebellum was not involved in smoothly produced tapping (Spencer et al., 2007). In sum, since the Robertson et al. (1999) studies, a number of other studies have consistently supported the idea of emergent timing as a distinct mode of timing.

The specific conditions under which an individual will use event-based or emergent timing are largely unclear. Until recently, it was thought that the style of movement (discrete, with distinct start and stop landmarks, or smoothly produced) determined which timing process (event-based or emergent) was engaged (Zelaznik et al., 2002), but there is some evidence that the presence of a regularly occurring sensory event may engage event-based timing even for tasks performed with smoothly produced movement (Zelaznik and Rosenbaum, 2010; Studenka and Zelaznik, 2011; Studenka et al., 2012). For example, hearing an auditory tone after completing a cycle of circle drawing can “eventize” timing behavior (Zelaznik and Rosenbaum, 2010). Delignières and Torre (2011) demonstrated that, while the two modes of timing are mutually exclusive in the performance of a task, individuals can alternate between event-based and emergent timing on the same task. It has been suggested that rate (Huys et al., 2008), and learning and practice, including experience with a given mode of timing, (Studenka and Zelaznik, 2008; Studenka et al., 2012) could play roles in determining which mode of timing is used.

In sum, previous research supports the existence of two separable and mutually exclusive timing modes but the conditions under which a specific mode is engaged are not clear. We know that musicians perform better than non-musicians on the event-based finger-tapping task and that musical training focuses largely on discrete movements, although smoothly produced movement may be a component of training depending on instrument type. To the best of our knowledge, musicians have not been compared to non-musicians on both discrete and continuous tasks. In the present experiment, we hypothesized that if event-based and emergent timing modes are dissociable, then musical training should be predominantly associated with superior temporal control in finger tapping. If musicians also perform better than non-musicians on circle drawing, this would suggest that either the two modalities are less separable than previously thought, or that musical training affects both event-based and emergent timing. To test this, we assessed performance on these tasks by measuring variability in the temporal domain, the smoothness of movement in the spatial domain and the relationship between the two.

## Materials and methods

### Participants

Twenty-two musicians (seven males) and seventeen non-musicians (four males) were recruited through the departments of Psychology and Music of local universities in Montréal, QC, as well as in between late night sets at various local jazz clubs. The age of musicians (*M* = 23 years, range = 18–33 years) and non-musicians (*M* = 22.2 years, range = 19–31 years) did not differ significantly [*t*_(37)_ = −0.657, *p* > 0.05]. Psychology undergraduate students were compensated with a one per cent credit applicable to a course grade. All other participants received 15 dollars. The study was approved by the Concordia University Human Research Ethics Committee and informed consent was given by all participants, who were debriefed about the goals of the experiment following their testing.

Non-musicians had minimal musical training (*M* = 2.2 years, *SD* = 1.6) and none were currently practicing. Musicians played a range of instruments, including guitar, piano, and violin, were all currently practicing and had at least 6 years of musical training (*M* = 13.9 years, *SD* = 4.3). All participants were strongly right-handed (*M* = 9.59, *SD* = 0.67 for musicians and *M* = 9.71, *SD* = 0.47 for non-musicians), as evaluated using the Edinburgh Handedness Inventory (Oldfield, 1971). Data from two additional non-musician participants were excluded based on poor performance of more than three standard deviations from the mean Paced or Unpaced IRI.

### Apparatus

Motion was recorded with the Visualeyez VZ3000 3D motion tracking system, manufactured by Phoenix Technologies. The markers consisted of infrared light emitting diodes (LED), each mounted on hard round plastic casing of 0.5 cm diameter and attached by thin copper wire to a central controller. For the finger tapping task, a single marker was affixed with Velcro tape to the nail of the right index finger. For the circle-drawing task, three markers were attached with Velcro tape to the surface of a pen of diameter 1.1 cm. The pen had a rounded plastic tip that participants used to trace circles. The LED wires had sufficient slack to allow for complete freedom of movement. Infrared-sensitive cameras tracked the position of the markers in three-dimensional space at a sampling rate of 200 Hz and to a spatial resolution of 0.015 mm. A National Instruments 6221 Data Acquisition board was used to synchronize the Visualeyez system with a computer-generated 1 KHz 20 ms metronome tone. Participants heard the metronome tone through a pair of Sony MDR-7506 headphones. Each participant was seated on a chair with independently adjustable seat and armrest heights.

Participants tapped and drew on a table of height 70.0 cm. For the circle drawing task, they traced a circle (7.0 cm diameter, 0.5 cm line thickness) printed in black on a white sheet of paper covered in a clear plastic sleeve affixed securely to the table top. A smaller (1.0 cm diameter) circle was printed on the larger circle's perimeter at the 12 o'clock position and served as the spatial target which participants were instructed to move the pen through in synchrony with the metronome.

### Stimuli, task, conditions, and procedure

Participants tapped and drew at four different rates (400, 550, 700, and 850 ms) for a total of eight different conditions, the order of which was counterbalanced across subjects such that tapping and drawing conditions were intermixed as determined by a Latin Square design. We tested across a range of metronome rates to assess the rate of change in IRI variability as a function of interval duration. The fastest rate at which both musician and non-musician participants could perform both tasks was determined by pilot testing. The slowest rate was set well below the limit of 1800 ms beyond which sensorimotor synchronization breaks down (Repp, 2005). Each condition consisted of six trials of 30 cycles of tapping or drawing paced by an auditory metronome. This was followed by sufficient time to complete 30 cycles of Unpaced tapping or drawing, assuming that a participant stayed reasonably close to the target rate. A final 1 KHz 20 ms tone indicated the end of each trial. All trials of a condition were performed one after another with a rest period of 30 s in between each trial.

For the tapping task, participants were told to tap their right index finger in synchrony with the metronome and use their full range of motion, without moving any other part of their body. They were then told that once the metronome stopped, they should continue tapping at the same pace until hearing the final tone. For the circle drawing task, participants were instructed to hold the pen in their right hand and trace the 7.0 cm circle template in the counterclockwise direction without stopping and such that the pen passed over the smaller 1.0 cm circle on the beat of the metronome. Identically to the tapping task, they were instructed to keep tracing at the same rate after the metronome stopped. At the beginning of the session, single practice trials of tapping and of circle drawing at 425 ms (a pace not used during the real test session) were administered to familiarize participants with the task.

Participants were also administered the Musical Experience Questionnaire (Bailey and Penhune, 2010) to assess age of initiation of musical training, years of musical training and hours of current practice. They were also given the Digit Symbol subtest of the Wechsler Adult Intelligence Scale-III (Wechsler, 1997) in order to evaluate processing speed and visuomotor coordination (Kaplan et al., 1991; Joy et al., 2003). Finally, they were given the Grooved Pegboard test (Lafayette Instrument Company) of fine motor control (Matthews and Klove, 1964). A testing session lasted approximately 2 h.

### Data analysis

#### Preprocessing

All temporal and kinematic measures were derived from the motion capture data synchronized with the digital metronome data. The data were analyzed using custom software written in Matlab (release R2007b, The MathWorks). The motion capture system permitted us to arbitrarily set a local coordinate reference frame and we did so using the tabletop as the *xy*-plane. This allowed us to examine the *z*-coordinate for finger height in tapping and the *x* and *y* coordinates for drawing.

To automatically detect cycle end points for tapping, we first filtered the motion capture data using a forward and reverse second-order low-pass Butterworth filter with a cutoff set at 50 Hz. The reverse filter compensated for any time shifts that might have been introduced by the forward filter. A high cut-off value allowed us to filter out noise inherent in the motion capture signal without removing the natural jitter of finger motion. Next, all local maxima, corresponding to the peak finger position for each cycle, were identified through an iterative search method. The acceleration curve was then calculated from the position curve and, next, the points of maximum acceleration between successive peaks were identified. These local maxima on the acceleration curve were prominent in all data, despite individual variations in tapping style, and they corresponded closely to the points at which the finger hit the tabletop because of the rapid change in velocity. In a final step, each point on the position curve that corresponded to a local acceleration maximum was automatically adjusted so that it coincided with the closest local minimum on the position curve. Thus, each identified tapping point closely coincided with the point of initial finger compression on the tabletop.

Circle drawing data was filtered using a forward and reverse second-order low-pass Butterworth filter with a cutoff set at 7 Hz (Roithner et al., 2000) in order to more easily detect cycle end points (the 12 o'clock position) in smoothed data. The trajectory data were then linearly transformed such that the origin of the coordinate reference frame was translated to the centre of the circle template. Then all points in the trajectory where the *x* coordinate changed sign from positive to negative (negative zero crossings) were identified as the cycle end points, where the pen crossed the anchor point at the top of the circle. To be considered valid crossings, the pen had to be on the table as indicated by the *z*-coordinate.

Once all tapping and drawing cycle endpoints were identified, further analysis focused on the Unpaced phases of tapping and drawing. The IRIs were calculated as the duration in time between adjacent points. For this and for all other measures, the first 2 cycles of the Unpaced phase of a trial were not used in the analysis (Zelaznik and Rosenbaum, 2010). Trials with fewer than 20 consecutive successfully identified cycle endpoints were excluded from analysis. We excluded 0.76% and 0.98% of tapping sequences for musicians and non-musicians, respectively. In the case of circle drawing, 4.17% of musicians' sequences and 3.68% of non-musicians' sequences were excluded. Amongst those sequences included in the analysis, the average sequence lengths for tapping (26.7 for musicians; 27.4 for non-musicians) and circle drawing (26.6 for musicians; 26.7 for non-musicians) were close to the maximum possible length, indicating that the detection algorithm successfully identified most taps and draws.

#### Basic performance measures

The coefficient of variation (standard deviation divided by the mean) was calculated for the Unpaced IRI's of each trial. The average coefficient of variation was then calculated across all trials of a condition for a given individual. This measure provides an overall view of the combination of all sources of IRI variability, including long-term drift away from the target tempo and motor, as well as timer, variability. To better isolate timer variability, we next removed linear drift from the time series of responses for each trial and examined the remaining variance. We carried out mixed-type ANOVA analyses for all dependent variables, using musical training as the between-groups variable and task and rate as within-subjects variables. We used the Bonferroni correction for multiple comparisons.

#### Analysis of timing modes and kinematics

To further analyze the IRI variability, we employed slope analysis (Ivry and Hazeltine, 1995). It can be shown that by plotting the variance against the square of the interval duration, the slope of the resulting line gives an estimate of the variance that is related to timing and the intercept is related to variability independent of interval duration, such as that stemming from execution of the motor response. Therefore, if two tasks share a common timing process, then the slopes related to each task should be equal, or at least correlated, within individuals. Equality of slopes is a necessary but not a sufficient condition for commonality of timing processes across tasks. However, if tapping and drawing slopes are unequal or, at the very least, uncorrelated, and if musical expertise affects only tapping slopes but not drawing slopes, this would be strong evidence in support of distinct timing processes. We used *t*-tests to assess the significance of slope correlations.

Another approach to demonstrating that tapping and drawing are associated with distinct timing processes is to determine whether or not task performance adheres to the Wing-Kristofferson open-loop model of event-based timing (Wing and Kristofferson, 1973), which partitions the response variability into two independent components: central (clock) and peripheral (motor). It follows from their model that the lag one covariances of the time series of responses will be negative (Zelaznik and Rosenbaum, 2010). In contrast, emergent timing is associated with a non-negative lag one covariance (Delignières and Torre, 2011). Therefore, the lag one covariance offers a means of verifying the kind of timing mode used in the execution of a task. To that end, we calculated the lag one covariance for the detrended IRI time series (Zelaznik and Rosenbaum, 2010).

We also examined the kinematic data with the goal of measuring the smoothness of movement. The more continuous motion is, the smoother we would expect it to be and a common measure of smoothness is mean squared jerk (Flash and Hogan, 1985). Mean squared jerk calculations were extracted from the motion capture data filtered at 50 Hz—the original circle data were re-filtered at this threshold. The normalized mean squared jerk was calculated per cycle by taking half the integral of squared jerk and multiplying this by a normalizing factor of duration to the fifth power divided by distance squared (Teulings et al., 1997). The square root of this value was used as the per-cycle normalized mean squared jerk. The average normalized mean squared jerk for a single trial was calculated from the per-cycle values.

## Results

### Cognitive measures

On the Digit Symbol test, musicians (*M* = 101.2; *SD* = 14.0) scored significantly higher than non-musicians (*M* = 92.1; *SD* = 10.1), *t*_(37)_ = −2.260, *p* = 0.030. Musicians were also faster (*M* = 53.9 s; *SD* = 4.3) than non-musicians (*M* = 59.0 s; *SD* = 9.0) when using their dominant (right) hand to complete the Grooved Pegboard test, *t*_(37)_ = 2.377, *p* = 0.023. These results suggest that the sample of musicians possessed superior fine motor control compared to the sample of non-musicians.

### Temporal measures

To determine if participants were able to carry out both tasks, we measured the mean IRI of the Paced and Unpaced phases separately for all conditions (Table 1). Mean IRI values were close to the target interval durations for both Paced and Unpaced phases, indicating that both groups of participants were generally able to produce accurately timed movements with and without the aid of a metronome. A 2 (musical training) × 2 (phase) × 2 (task) × 4 (rate) repeated measures ANOVA of mean IRI revealed an interaction of phase and task [*F*_(1, 37)_ = 11.882, *p* < 0.01] such that participants generally had shorter Paced IRI's for drawing compared to tapping (*p* = 0.01). Furthermore, participants' mean IRIs for tapping were shorter in the Unpaced compared to Paced phases (*p* = 0.017). However, there was no main effect of musical training nor were there any significant interactions involving musical training. In sum, there were no differences in the mean IRI of either the Paced or Unpaced phases that were related to musical training.

**Table 1:** **Mean inter-response intervals of the Paced and Unpaced phases**.

	**Prescribed interval duration**							
	**400 ms**		**550 ms**		**700 ms**		**850 ms**	
	**Paced**	**Unpaced**	**Paced**	**Unpaced**	**Paced**	**Unpaced**	**Paced**	**Unpaced**
**MUSICIANS**								
Tapping	400.06 (0.93)	401.79 (10.06)	549.95 (1.10)	550.47 (14.23)	699.85 (0.95)	689.74 (16.00)	847.34 (10.82)	844.97 (34.53)
Drawing	395.53 (13.63)	407.16 (12.82)	538.04 (16.31)	544.17 (19.20)	687.30 (9.82)	686.48 (19.88)	836.70 (16.31)	837.09 (34.68)
**NON-MUSICIANS**								
Tapping	395.31 (12.09)	390.54 (22.55)	548.52 (3.86)	539.68 (22.41)	697.96 (2.68)	675.18 (34.88)	846.61 (5.02)	827.05 (51.72)
Drawing	396.53 (20.96)	408.16 (25.20)	541.3 (23.63)	546.95 (28.38)	684.28 (43.51)	679.22 (56.36)	841.52 (36.58)	837.74 (41.47)

Note: All values in milliseconds. Standard deviations in parentheses.

**Figure 1:**
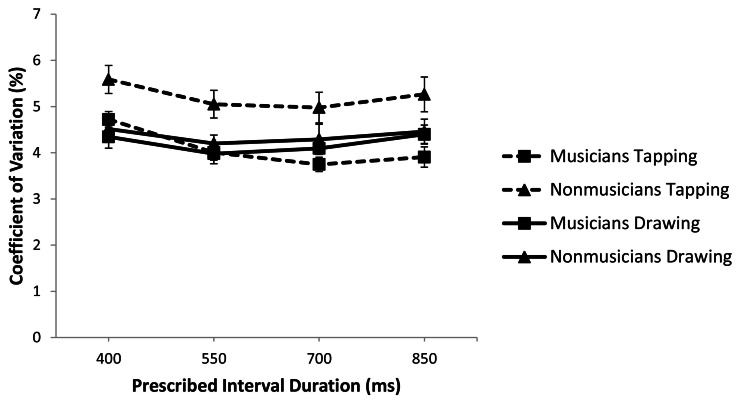
**Coefficient of variation of the Unpaced IRI, plotted against prescribed interval duration and with standard error bars**.

As a first step in assessing variability of performance in the Unpaced phase, we compared the coefficient of variation of the IRI for musicians and non-musicians across tasks and rates using a 2 (musical training) × 2 (task) × 4 (rate) repeated measures ANOVA (Figure 1). There was a significant interaction between task and musical expertise [*F*_(1, 37)_ = 10.207, *p* < 0.05; *partial* η^2^ = 0.216] such that non-musicians were significantly more variable in tapping compared to musicians (*p* < 0.01) but the two groups did not differ in drawing (*p* = 0.454). Furthermore, while non-musicians were more variable in tapping than in drawing (*p* < 0.01), musicians did not differ on these tasks (*p* = 0.587).

To better isolate timer variability we analyzed the variance of the Unpaced IRI sequences after linear detrending. Musicians were less variable than non-musicians overall [*F*_(1, 37)_ = 8.691, *p* < 0.01, *partial* η^2^ = 0.190]. For both groups, variability increased with interval duration [*F*_(3, 111)_ = 88.32, *p* < 0.01; *partial* η^2^ = 0.705], consistent with previous studies (Robertson et al., 1999). Consistent with the analysis of the coefficient of variation, there was an interaction between task and musical expertise [*F*_(1, 37)_ = 7.325, *p* < 0.05; *partial* η^2^ = 0.165]. Pairwise comparisons showed that musicians were less variable than non-musicians for tapping (*p* < 0.01) but not for drawing (*p* = 0.425). Furthermore, while non-musicians were more variable in tapping than in drawing (*p* < 0.01), musicians did not differ between these two tasks (*p* = 0.421). Taken together, the results of both the coefficient of variation and the detrended variance analyses suggest that musical training is associated with greater regularity of Unpaced tapping but has no effect on Unpaced drawing.

**Figure 2:**
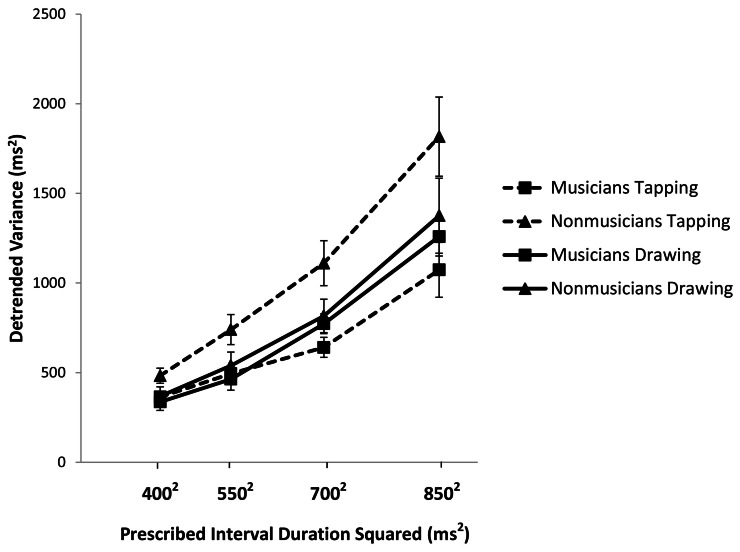
**Detrended variance of the Unpaced IRI plotted against the square of the prescribed interval duration and with standard error bars**.

**Figure 3:**
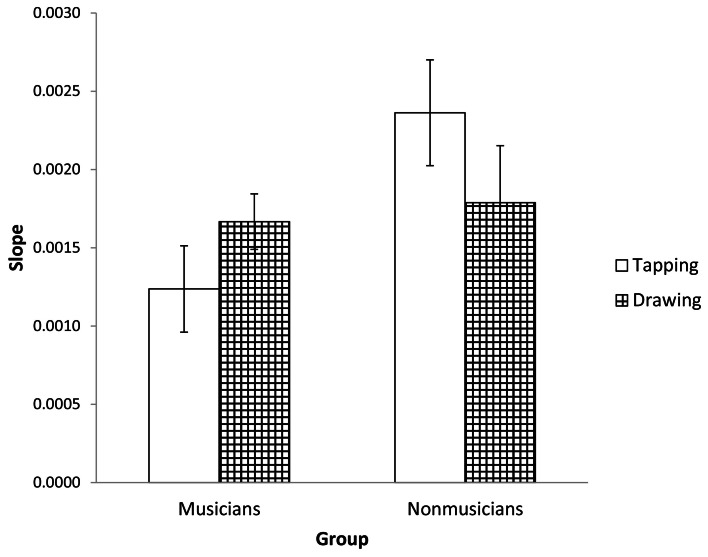
**Average slopes of the lines formed from plotting Unpaced IRI variance against the square of the prescribed interval duration.** Standard error bars are shown.

It may be the case that the observed differences in variability are due to better motor control by musicians in the tapping task, but that underlying timing processes are unaffected by expertise. To test this possibility, we used slope analysis to partition the IRI variability into timer-related and non-timer-related (e.g., motor) components (Ivry and Hazeltine, 1995; Robertson et al., 1999). Timer variability is expected to increase with IRI, resulting in positive slope values across rates (Robertson et al., 1999). A 2 (group) by 2 (task) repeated measures ANOVA for the slope values showed a marginally significant interaction between task and musical training [*F*_(1, 37)_ = 3.940, *p* = 0.055, *partial* η^2^ = 0.096]. Pairwise comparisons showed that musicians had significantly smaller slopes than non-musicians for tapping (*M* = 0.00124 and 0.00236 respectively; *p* < 0.05; see Figures 2 and 3) but there were no differences between groups for drawing (*M* = 0.00167 and 0.00179 respectively; *p* = 0.75). Within groups, there were no statistically significant differences between tasks. For both musicians and non-musicians, tapping and drawing slopes were not correlated, suggesting that these tasks were using unrelated timing processes (Ivry and Hazeltine, 1995). Overall, these results further suggest that musical training only affected timing for the tapping task.

**Figure 4:**
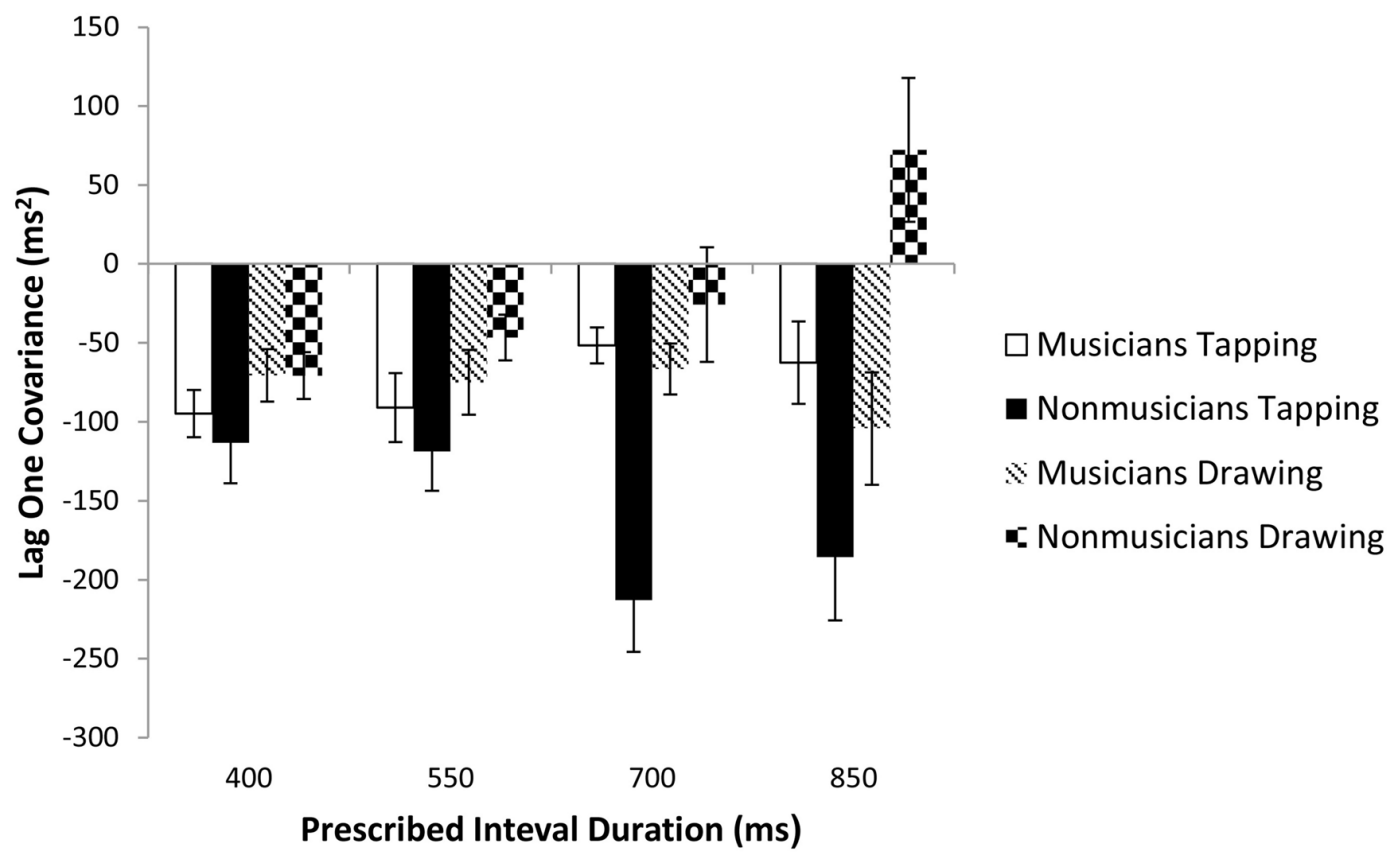
**Lag one covariances of the IRI for the four prescribed interval durations.** Standard error bars are shown.

To further investigate the timing processes used by each group on the tapping and drawing tasks, we examined whether performance on each task adhered to the Wing and Kristofferson (1973) event-based model of timing. The Wing-Kristofferson model predicts significantly negative lag one covariances for tapping (Wing and Kristofferson, 1973; Zelaznik and Rosenbaum, 2010). In contrast, emergent timing is characterized by non-negative lag one covariance (Delignières and Torre, 2011). A series of one-sample *t*-tests were carried out on individual lag values with a significance level set at 0.01 to correct for multiple comparisons (Zelaznik and Rosenbaum, 2010). They revealed that most lag one covariances were negative (Figure 4), with the exception of musicians tapping at 850 ms (*p* = 0.026) and non-musicians drawing at 700 and 850 ms (*p* = 0.486 and 0.131 respectively). Overall these results suggest that musicians were largely using event-based timing for tapping and drawing while non-musicians were engaging event-based timing processes for tapping and emergent timing processes for drawing.

### Kinematic measures

**Figure 5:**
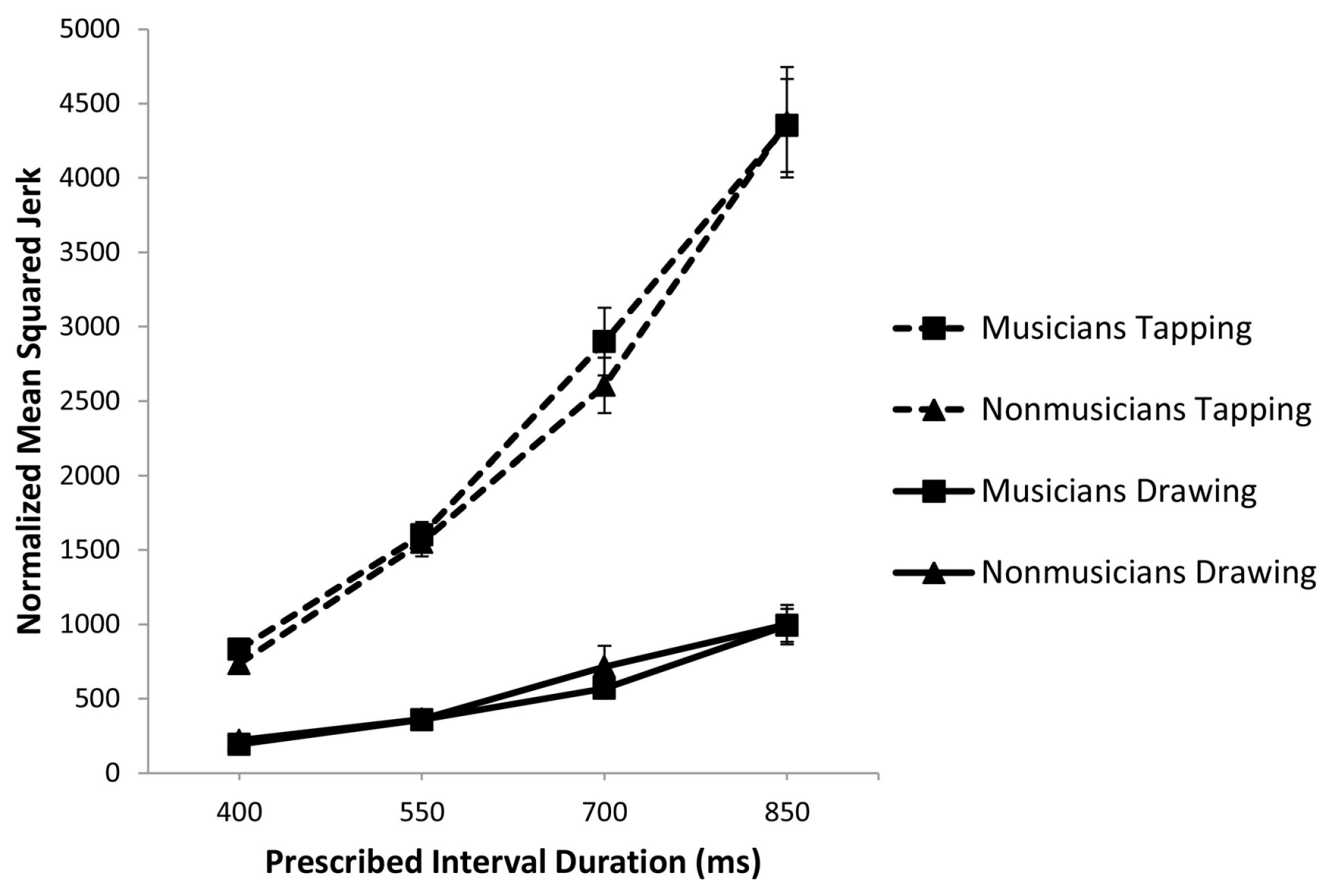
**Normalized mean squared jerk plotted against prescribed interval duration and with standard error bars**.

To analyze smoothness of movement, we compared the average normalized mean squared jerk per cycle between groups for a given task (Zelaznik and Rosenbaum, 2010). For tapping, we examined mean squared jerk of the *z* coordinate of movement and for circle drawing, we analyzed mean squared jerk in the *x* (3 o'clock to 9 o'clock) direction. A 2 (musical training) × 2 (task) × 4 (rate) repeated measures ANOVA showed a main effect of task [*F*_(1, 111)_ = 272.311, *p* < 0.001, *partial* η^2^ = 0.880] such that tapping is significantly less smooth than circle drawing (Figure 5). There was a main effect of rate [*F*_(3, 111)_ = 244.019, *p* < 0.001] but no effect of musical training (*p* = 0.640) or interaction between musical training and either task (*p* = 0.498) or rate (*p* = 0.966). In sum, tapping was significantly jerkier than circle drawing and musicians and non-musicians did not differ in their movement smoothness for either task.

We examined the relationship between an individual's movement kinematics and their timing variability by analyzing the bivariate correlations between mean squared jerk and detrended IRI variance (Table 2). It has been suggested that jerkiness aids timing in a tapping task (Balasubramaniam et al., 2004). Therefore we expected to find negative correlations between jerkiness and IRI variability for tapping. In the case of circle drawing, given that timing emerges from movement once kinematic parameters are stabilized, it seemed reasonable to expect that the less jerky movement becomes, the less variable timing would be. Musicians exhibited no significant correlations between jerkiness and IRI variability for either task. However, non-musicians displayed significant or marginally significant negative correlations at the three slowest tapping rates, suggesting that jerkier movement is associated with decreased IRI variability in tapping. On the other hand, when it came to drawing, non-musicians had significant positive correlations between jerk and IRI variability at most rates. The same pattern of correlations was found for the drawing conditions when basing the jerk calculations on either the X or Y coordinate of movement. To sum up, musicians unexpectedly showed no relationship between jerk and IRI variability, while non-musicians showed a negative association for tapping and a positive association for drawing.

**Table 2:** **Correlations between normalized mean squared jerk (*Z*-coordinate for tapping; *X* coordinate for drawing) and detrended IRI variance**.

	**Prescribed interval duration**							
	**Tapping**				**Drawing**			
	**400 ms**	**550 ms**	**700 ms**	**850 ms**	**400 ms**	**550 ms**	**700 ms**	**850 ms**
Musicians	−0.217	−0.345	0.309	0.022	−0.003	−0.266	−0.299	−0.006
Non-musicians	−0.586^*^	−0.461^+^	−0.456^++^	−0.310	0.608^*^	0.540^*^	0.624^*^^+++^	0.103

Note:

* p < 0.05;

+ p = 0.063;

++ p = 0.066;

+++ after removing a single outlier.

## Discussion

The main purpose of this study was to test the dissociability of event-based and emergent timing mechanisms in trained musicians. We know that musicians excel at tasks that typically engage event-based timing, such that musical training has been associated with greater temporal accuracy and precision in rhythmic tapping tasks (Franěk et al., 1991; Repp, 2010; Bailey and Penhune, 2012). We therefore reasoned that if the two modes of timing are dissociable, then expertise in predominantly event-based timing would not affect performance on tasks associated with emergent timing. We investigated this by examining both temporal and kinematic measures of performance for finger tapping and circle drawing.

Consistent with our hypothesis that event-based and emergent timing are dissociable, we found that musical training affects temporal variability in tapping but not drawing. This result is borne out across three different measures. When we examined the coefficient of variation of the IRI, the detrended IRI variance, and the variance related exclusively to timing as estimated from a slope analysis, we consistently found that musicians were less variable than non-musicians in tapping but did not differ in drawing. This set of results suggests that the effects of musical training do not transfer to continuous circle drawing and lends support to the hypothesis that event-based and emergent timing are dissociable processes.

Another piece of evidence supporting the dissociability of the two timekeeping processes is that the sign of the relationship between movement jerk and timing variability appears to be a function of task, but only for non-musicians. It has been suggested that greater jerk may result in a greater volume of proprioceptive information which may aid in maintaining the regularity of movement in an event-based task (Balasubramaniam et al., 2004). In our analysis, the control group of non-musicians generally showed a negative correlation between jerk and IRI variance for tapping, as expected. However, musicians did not show any relationship between movement jerk and IRI variance for either task. It may be that the event-based timing of musicians is precise and accurate enough that the additional information garnered from jerky movement is not needed to successfully perform event-based timing tasks. Indeed, our slope analysis of tapping showed that the timer-related variance of musicians was only 52% of that of non-musicians. Thus, the difference between musicians and non-musicians in how timing related to movement in tapping could be explained by the more precise timekeeping of musicians.

In a repetitive, smoothly produced movement task that uses emergent timing, less jerk indicates more regular movement which should lead to less variability in timing if timing emerges from movement kinematics. Once again, musicians did not show any relationship between jerk and IRI variance but non-musicians generally showed the expected positive correlation for drawing. Although musicians were not better than non-musicians at the drawing task, our results indicate that musicians did not rely on a kinematic strategy, similar to their results for tapping. In sum, musical training was associated with a decoupling of movement jerk and IRI variability for both tasks. These group differences for tapping and drawing suggest that any relationships that may exist between kinematics and timing are malleable through experience and not a requirement of the timing system.

The analyses of IRI variability discussed so far help to determine if timing modes are the same or different across tasks. The lag one covariance of the IRI can help us to identify which mode of timing is being used in a given task. Lag one covariances are predicted to be negative for event-based timing and nonnegative for emergent timing (Wing and Kristofferson, 1973; Zelaznik and Rosenbaum, 2010; Delignières and Torre, 2011). In the present study, analysis of the lag one covariances revealed additional differences in how musicians and non-musicians executed both tasks. Lag one covariances were almost uniformly negative for musicians across both tasks, suggesting that they were using event-based timing for tapping and drawing. This result is inconsistent with the results of the slope analysis for musicians, which did not yield any significant correlation between slopes for these two tasks, implying the use of different timing modes in the two tasks. Non-musicians exhibited lag one covariances that were negative for tapping and nonnegative for drawing at the two slowest rates. This pattern of results is closer to the expected patterns for event-based tapping and emergent drawing.

What could account for the inconsistency between musicians' lag one IRI covariances and their IRI variance slopes? Using lag one covariances to identify the mode of timing comes with a risk of misidentifying event-based timing as emergent (Lemoine and Delignières, 2009). This caveat may not apply to the present study, for which a misidentification of emergent timing as event-based seems the more likely scenario. Another possible explanation is that a relationship between tapping and drawing slopes for musicians is being suppressed by some other factor. For musicians with greater than the median amount of 13.5 years of experience there was a marginally significant correlation between tapping and drawing slopes (*r* = 0.570, *p* = 0.067). We speculate that the most experienced musicians in our study may have been using event-based timing for circle drawing but additional experiments with a larger sample size and a more uniform distribution of years of musical training are needed to resolve this issue.

If a subset of our musician group did use event-based timing on the drawing task, then it would be consistent with recent studies that show that priming effects may play a role in the use of event-based timing when emergent timing would normally be used. When intermixing tapping and drawing conditions in a fixed order for all subjects, unexpected significant correlations of the IRI coefficient of variation have been observed between the final circle drawing condition that was expected to exhibit emergent timing and previous conditions that were event-like (Zelaznik and Rosenbaum, 2010; Studenka et al., 2012). It has been suggested that the previous event-timed conditions in the fixed ordering led to a practice effect that primed participants to use event-based timing on the final condition that might otherwise have shown the signature of emergent timing (Studenka et al., 2012). Similarly, in the present study, the event-based timing that at least some musicians may be using for circle drawing could be the result of years of extensive musical training—long-term practice effects that primed participants to use event-based timing.

In general, a target for future study is the identification of the aspects of musical training that are responsible for the relationships observed in the present study. Intensity of musical practice, rather than total years of musical training, has been found to be associated with superior ability to improve tactile discrimination in the index fingers of pianists (Ragert et al., 2004). In a comparison of percussionists, pianists, singers, and non-musicians, the type of musical instrument was found to affect timing variability such that drummers were the least variable (Krause et al., 2010). In the present study, we did not have a sufficiently large sample size to address these issues. These or other parameters of musical experience, such as the age of onset of training (Bailey and Penhune, 2012) could be contributing to the results of the present study and could become the focus of future studies.

Our study is novel for its comparison of musicians and non-musicians on event-based and emergent timing tasks. Most importantly, our results add to the body of research supporting the dissociability of event-based and emergent timing. Our results also suggest that the effects of musical training on timer variability may be limited to the kinds of tasks and modes of timing used in musical performance. They also demonstrate that the relationship between movement and timing may depend on experience. Further investigation is needed to identify the aspects of musical training that contribute to these differences between musicians and non-musicians.

### Conflict of interest statement

The authors declare that the research was conducted in the absence of any commercial or financial relationships that could be construed as a potential conflict of interest.

## References

[B1] Bailey J. A. Penhune V. B. (2010). Rhythm synchronization performance and auditory working memory in early- and late-trained musicians. Exp. Brain Res. 204, 91–101. 10.1007/s00221-010-2299-y 20508918

[B2] Bailey J. Penhune V. B. (2012). A sensitive period for musical training: contributions of age of onset and cognitive abilities. Ann. N.Y. Acad. Sci. 1252, 163–170. 10.1111/j.1749-6632.2011.06434.x 22524355

[B3] Balasubramaniam R. Wing A. M. Daffertshofer A. (2004). Keeping with the beat: movement trajectories contribute to movement timing. Exp. Brain Res. 159, 129–134. 10.1007/s00221-004-2066-z 15365663

[B4] Collier G. L. Ogden R. T. (2004). Adding drift to the decomposition of simple isochronous tapping: an extension of the Wing-Kristofferson model. J. Exp. Psychol. Hum. Percept. Perform. 30, 853–872. 10.1037/0096-1523.30.5.853 15462625

[B5] Delignières D. Torre K. (2011). Event-based and emergent timing: dichotomy or continuum? A reply to Repp and Steinman (2010). J. Mot. Behav. 43, 311–318. 10.1080/00222895.2011.588274 21774607

[B6] Flash T. Hogan N. (1985). The coordination of arm movements: an experimentally confirmed mathematical model. J. Neurosci. 5, 1688–1703. 4020415 10.1523/JNEUROSCI.05-07-01688.1985 PMC6565116

[B7] Franěk M. Mates J. Radil T. Beck K. Pöppel E. (1991). Finger tapping in musicians and nonmusicians. Int. J. Psychophysiol. 11, 277–279. 1797762 10.1016/0167-8760(91)90022-p

[B8] Huys R. Studenka B. E. Rheaume N. L. Zelaznik H. N. Jirsa V. K. (2008). Distinct timing mechanisms produce discrete and continuous movements. PLoS Comput. Biol. 4:e1000061. 10.1371/journal.pcbi.1000061 18437236 PMC2329590

[B9] Ivry R. B. Hazeltine R. E. (1995). Perception and production of temporal intervals across a range of durations: evidence for a common timing mechanism. J. Exp. Psychol. Hum. Percept. Perform. 21, 3–18. 10.1037/0096-1523.21.1.3 7707031

[B10] Ivry R. B. Keele S. W. Diener H. C. (1988). Dissociation of the lateral and medial cerebellum in movement timing and movement execution. Exp. Brain Res. 73, 167–180. 3208855 10.1007/BF00279670

[B11] Joy S. Fein D. Kaplan E. (2003). Decoding digit symbol: speed, memory, and visual scanning. Assessment 10, 56–65. 10.1177/0095399702250335 12675384

[B12] Kaplan E. Fein D. Morris R. Delis D. (1991). WAIS-R as a Neuropsychological Instrument. San Antonio, TX: The Psychological Corporation.

[B13] Krause V. Pollok B. Schnitzler A. (2010). Perception in action: the impact of sensory information on sensorimotor synchronization in musicians and non-musicians. Acta Psychol. 133, 28–37. 10.1016/j.actpsy.2009.08.003 19751937

[B14] Lemoine L. Delignières D. (2009). Detrended windowed (lag one) autocorrelation: a new method for distinguishing between event-based and emergent timing. Q. J. Exp. Psychol. (Hove) 62, 585–604. 10.1080/17470210802131896 18609399

[B15] Matthews C. G. Klove H. (1964). Instruction Manual for the Adult Neuropsychology Test Battery. Madison: University of Wisconsin Medical School.

[B16] Oldfield R. C. (1971). The assessment and analysis of handedness: the Edinburgh inventory. Neuropsychologia 9, 97–113. 5146491 10.1016/0028-3932(71)90067-4

[B17] Ragert P. Schmidt A. Altenmüller E. Dinse H. R. (2004). Superior tactile performance and learning in professional pianists: evidence for meta-plasticity in musicians. Eur. J. Neurosci. 19, 473–478. 10.1111/j.0953-816X.2003.03142.x 14725642

[B18] Repp B. H. (2005). Sensorimotor synchronization: a review of the tapping literature. Psychon. Bull. Rev. 12, 969–992. 16615317 10.3758/bf03206433

[B19] Repp B. H. (2010). Sensorimotor synchronization and perception of timing: effects of music training and task experience. Hum. Mov. Sci. 29, 200–213. 10.1016/j.humov.2009.08.002 20074825

[B20] Repp B. H. Doggett R. (2007). Tapping to a very slow beat: a comparison of musicians and nonmusicians. Music Percept. 24, 367–376.

[B21] Robertson S. D. Zelaznik H. N. Lantero D. A. Bojczyk K. G. Spencer R. M. Doffin J. G.. (1999). Correlations for timing consistency among tapping and drawing tasks: evidence against a single timing process for motor control. J. Exp. Psychol. Hum. Percept. Perform. 25, 1316–1330. 10.1037/0096-1523.25.5.1316 10531665

[B22] Roithner R. Schwameder H. Müller E. (2000). Determination of optimal filter parameters for filtering kinematic walking data using Butterworth low pass filter, in ISBS – Conference Proceedings Archive, 1. Retrieved from: https://ojs.ub.uni-konstanz.de/cpa/article/view/2197

[B23] Spencer R. M. C. Verstynen T. Brett M. Ivry R. B. (2007). Cerebellar activation during discrete and not continuous timed movements: an fMRI study. Neuroimage 36, 378–387. 10.1016/j.neuroimage.2007.03.009 17459731 PMC1905817

[B24] Spencer R. M. C. Zelaznik H. N. Diedrichsen J. Ivry R. B. (2003). Disrupted timing of discontinuous but not continuous movements by cerebellar lesions. Science 300, 1437–1439. 10.1126/science.1083661 12775842

[B25] Studenka B. E. Zelaznik H. N. (2008). The influence of dominant versus non-dominant hand on event and emergent motor timing. Hum. Mov. Sci. 27, 29–52. 10.1016/j.humov.2007.08.004 18191491

[B26] Studenka B. E. Zelaznik H. N. (2011). Synchronization in repetitive smooth movement requires perceptible events. Acta Psychol. 136, 432–441. 10.1016/j.actpsy.2011.01.011 21300324

[B27] Studenka B. E. Zelaznik H. N. Balasubramaniam R. (2012). The distinction between tapping and circle drawing with and without tactile feedback: an examination of the sources of timing variance. Q. J. Exp. Psychol. 65, 1086–1100. 10.1080/17470218.2011.640404 22332846

[B28] Teulings H. L. Contreras-Vidal J. L. Stelmach G. E. Adler C. H. (1997). Parkinsonism reduces coordination of fingers, wrist, and arm in fine motor control. Exp. Neurol. 146, 159–170. 10.1006/exnr.1997.6507 9225749

[B29] Turvey M. T. (1977). Preliminaries to a theory of action with reference to vision, in Perceiving Acting, and Knowing: Toward an Ecological Psychology, eds Shaw R. E. Bransford J. (Hillsdale, NJ: Erlbaum), 211–265.

[B30] Wechsler D. (1997). Wechsler Adult Intelligence Scale: WAIS-III. Manual. San Antonio, TX: The Psychological Corporation.

[B31] Wing A. M. (2002). Voluntary timing and brain function: an information processing approach. Brain Cogn. 48, 7–30. 10.1006/brcg.2001.1301 11812030

[B32] Wing A. M. Kristofferson A. B. (1973). Response delays and the timing of discrete motor responses. Percept. Psychophys. 14, 5–12. 11543514

[B33] Zelaznik H. N. Rosenbaum D. A. (2010). Timing processes are correlated when tasks share a salient event. J. Exp. Psychol. Hum. Percept. Perform. 36, 1565–1575. 10.1037/a0020380 20731516

[B34] Zelaznik H. N. Spencer R. M. C. Ivry R. B. (2002). Dissociation of explicit and implicit timing in repetitive tapping and drawing movements. J. Exp. Psychol. Hum. Percept. Perform. 28, 575–588. 10.1037/0096-1523.28.3.575 12075889

[B35] Zelaznik H. N. Spencer R. M. C. Ivry R. B. Baria A. Bloom M. Dolansky L.. (2005). Timing variability in circle drawing and tapping: probing the relationship between event and emergent timing. J. Mot. Behav. 37, 395–403. 10.3200/JMBR.37.5.395-403 16120566 PMC1904497

